# Sphingosine-1-phosphate lyase mutations cause primary adrenal insufficiency and steroid-resistant nephrotic syndrome

**DOI:** 10.1172/JCI90171

**Published:** 2017-02-06

**Authors:** Rathi Prasad, Irene Hadjidemetriou, Avinaash Maharaj, Eirini Meimaridou, Federica Buonocore, Moin Saleem, Jenny Hurcombe, Agnieszka Bierzynska, Eliana Barbagelata, Ignacio Bergadá, Hamilton Cassinelli, Urmi Das, GOSgene, Ruth Krone, Bulent Hacihamdioglu, Erkan Sari, Ediz Yesilkaya, Helen L. Storr, Maria Clemente, Monica Fernandez-Cancio, Nuria Camats, Nanik Ram, John C. Achermann, Paul P. Van Veldhoven, Leonardo Guasti, Debora Braslavsky, Tulay Guran, Louise A. Metherell

**Affiliations:** 1 Centre for Endocrinology, William Harvey Research Institute, John Vane Science Centre, Queen Mary, University of London, Charterhouse Square, London, United Kingdom.; 2 Genetics and Genomic Medicine, UCL Great Ormond Street Institute of Child Health, University College London, London, United Kingdom.; 3 Children’s and Academic Renal Unit, University of Bristol, Bristol, United Kingdom.; 4 Servicio de Nefrología, Hospital de Niños “Ricardo Gutiérrez,” Buenos Aires, Argentina.; 5 Centro de Investigaciones Endocrinológicas “Dr. Cesar Bergadá” (CEDIE) – CONICET – FEI – División de Endocrinología, Hospital de Niños “Ricardo Gutiérrez,” Buenos Aires, Argentina.; 6 Alderhey Children’s Hospital NHS Foundation Trust, Eaton Road, Liverpool, United Kingdom.; 7 The center is detailed in the Supplemental Acknowledgments.; 8 Birmingham Children’s Hospital, Birmingham, United Kingdom.; 9 Health Sciences University, Suleymaniye Maternity and Children’s Training and Research Hospital, Department of Paediatric Endocrinology and Diabetes, Istanbul, Turkey.; 10 Gulhane Military Medical School Department of Paediatric Endocrinology and Diabetes, Ankara, Turkey.; 11 Growth and Development Research Unit, Vall d’Hebron Research Institute (VHIR), Hospital Vall d’Hebron, CIBERER, Instituto de Salud Carlos III, Barcelona, Spain.; 12 Department of Medicine, Aga Khan University Hospital, Karachi, Pakistan.; 13 Laboratory of Lipid Biochemistry and Protein Interactions (LIPIT), Campus Gasthuisberg, KU Leuven, Leuven, Belgium.; 14 Marmara University, Department of Paediatric Endocrinology and Diabetes, Istanbul, Turkey.

## Abstract

Primary adrenal insufficiency is life threatening and can present alone or in combination with other comorbidities. Here, we have described a primary adrenal insufficiency syndrome and steroid-resistant nephrotic syndrome caused by loss-of-function mutations in sphingosine-1-phosphate lyase (SGPL1). SGPL1 executes the final decisive step of the sphingolipid breakdown pathway, mediating the irreversible cleavage of the lipid-signaling molecule sphingosine-1-phosphate (S1P). Mutations in other upstream components of the pathway lead to harmful accumulation of lysosomal sphingolipid species, which are associated with a series of conditions known as the sphingolipidoses. In this work, we have identified 4 different homozygous mutations, c.665G>A (p.R222Q), c.1633_1635delTTC (p.F545del), c.261+1G>A (p.S65Rfs*6), and c.7dupA (p.S3Kfs*11), in 5 families with the condition. In total, 8 patients were investigated, some of whom also manifested other features, including ichthyosis, primary hypothyroidism, neurological symptoms, and cryptorchidism. *Sgpl1^–/–^* mice recapitulated the main characteristics of the human disease with abnormal adrenal and renal morphology. *Sgpl1^–/–^* mice displayed disrupted adrenocortical zonation and defective expression of steroidogenic enzymes as well as renal histology in keeping with a glomerular phenotype. In summary, we have identified *SGPL1* mutations in humans that perhaps represent a distinct multisystemic disorder of sphingolipid metabolism.

## Introduction

Primary adrenal insufficiency (PAI) is most commonly congenital in children. Manifestations can include hyperpigmentation, failure to thrive, and a poor response to illness, with hypoglycemia and hypotension. Reduced life expectancy is described, and the condition can be fatal if undetected. It is genetically heterogeneous, with some gene defects causing syndromic disease. Mechanisms of disease include steroid biosynthetic defects, adrenocorticotropic hormone (ACTH) resistance, adrenal dysgenesis, cholesterol synthesis disorders, and metabolic disorders incorporating peroxisomal and mitochondrial defects (reviewed in refs. 1, 2). Our group has previously identified a number of genes in adrenal insufficiency syndromes (3–8); however, within our patient cohort (*n* > 350), the genetic cause is currently unknown for 38% of cases, rendering their prognosis uncertain.

**Figure 1:**
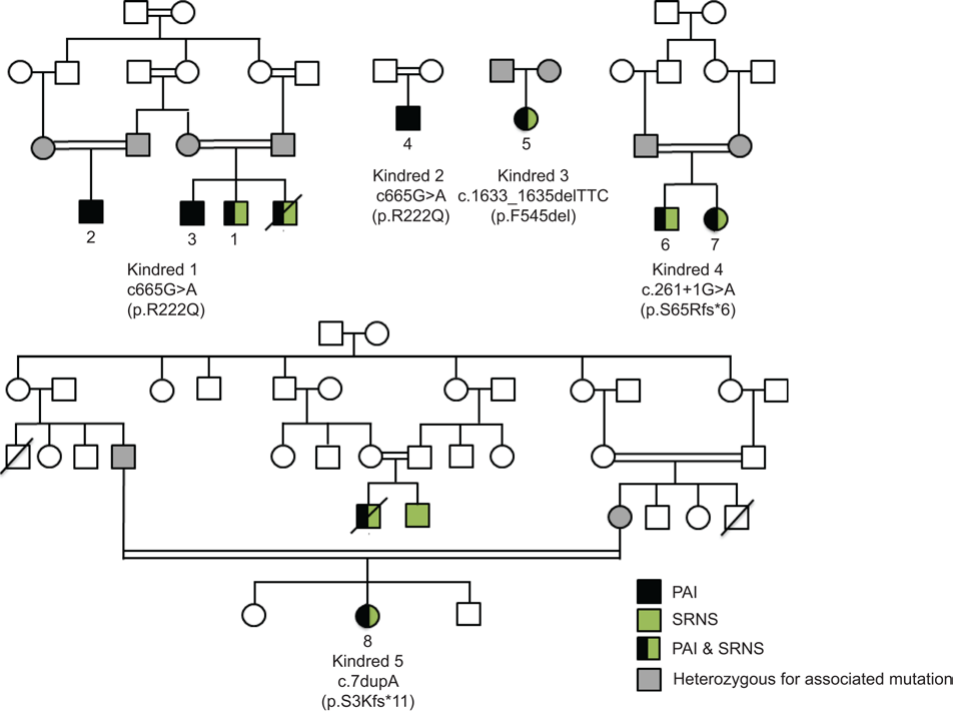
Pedigrees of kindreds 1 to 5 where all affected individuals manifested PAI, with or without SRNS, and were positive for mutations in *SGPL1*. Black symbols indicate individuals with PAI alone, half-filled in green indicate those who additionally had steroid-resistant nephrotic syndrome, and green indicate those with SRNS alone. All affected individuals were homozygous for the indicated mutations (patients sequenced have been numbered from 1 to 8), and parents were heterozygous (those sequenced denoted by gray symbols). Mutations for patients 2, 3, and 5 were identified by WES and the remainder by Sanger sequencing of *SGPL1*.

We investigated one extended, consanguineous kindred with 4 affected individuals with PAI, 2 of whom also had steroid-resistant nephrotic syndrome, described as focal segmental glomerulosclerosis (FSGS) (9) (Figure 1, kindred 1). Inheritance was suggestive of an autosomal recessive pattern, and we used whole-exome sequencing (WES) to investigate. Mutations identified in a candidate gene were then sought in the remainder of our cohort and in patients manifesting FSGS alone.

## Results

### Human genetic findings.

The index case, patient 1, was diagnosed with an isolated glucocorticoid deficiency at age 8 months, when he presented with genital hyperpigmentation. He subsequently developed steroid-resistant nephrotic syndrome with biopsy findings of FSGS at 2.5 years and received a kidney transplant at age 5 years (9). A younger sibling with similar clinical history (not sequenced) died at age 4 years, while an older sibling, at 8 years, and a cousin, at 3 years, only have PAI (9) (Figure 1, kindred 1; Table 1).

Affected individuals were mutation negative for the known genetic causes of PAI (3). WES was carried out for 2 affected individuals in the pedigree (patients 2 and 3; Figure 1). SNPs, with a threshold coverage of at least 10 reads on the respective nucleotides, were assessed using Ingenuity Variant Analysis (http://www.ingenuity.com/products/variant-analysis; detailed description in Methods). In brief, the number of variants was reduced by the following strategy: (i) identifying variants that were common to both individuals; (ii) excluding variants that were heterozygous; (iii) removing variants, annotated in SNP databases (dbSNP, http://www.ncbi.nlm.nih.gov/SNP/, release 85; Exome Aggregation Consortium [ExAC], http://exac.broadinstitute.org, accessed July 2016), with a minor allele frequency of greater than 0.01; and (iv) evaluating nonsynonymous coding variants, splice variants, and indels only. Finally, candidate variants in 2 genes *(*Chr10:69957117C>T in *MYPN* and Chr10:72628151G>A in *SGPL1*) were investigated for segregation with disease in the kindred by Sanger sequencing. Both variants segregated with disease in the family. *MYPN* encodes myopalladin, which is expressed in striated muscle and functions as a structural, signaling, and gene expression regulatory molecule in response to muscle stress. Variants in *MYPN* have previously been implicated in dilated cardiomyopathy, not a clinical finding in our patients, and although rare (allele count 69/121208), there are 2 homozygotes noted in ExAC for this variant. Thus, we demoted this variant in *MYPN* and focused on the variant in *SGPL1*. This variant, Chr10:72628151G>A c.665G>A; p.R222Q, has a minor allele frequency of 1.656e-5; this represents 2/120744 alleles reported in the ExAC browser. Both are heterozygotes; one is from the South Asian population and the other non-Finnish European. No homozygotes are annotated in any database (see dbSNP; ExAC; National Heart, Lung, and Blood Institute [NHLBI] Exome Sequencing Project [ESP], Exome Variant Server, http://evs.gs.washington.edu/EVS/)

**Figure 2:**
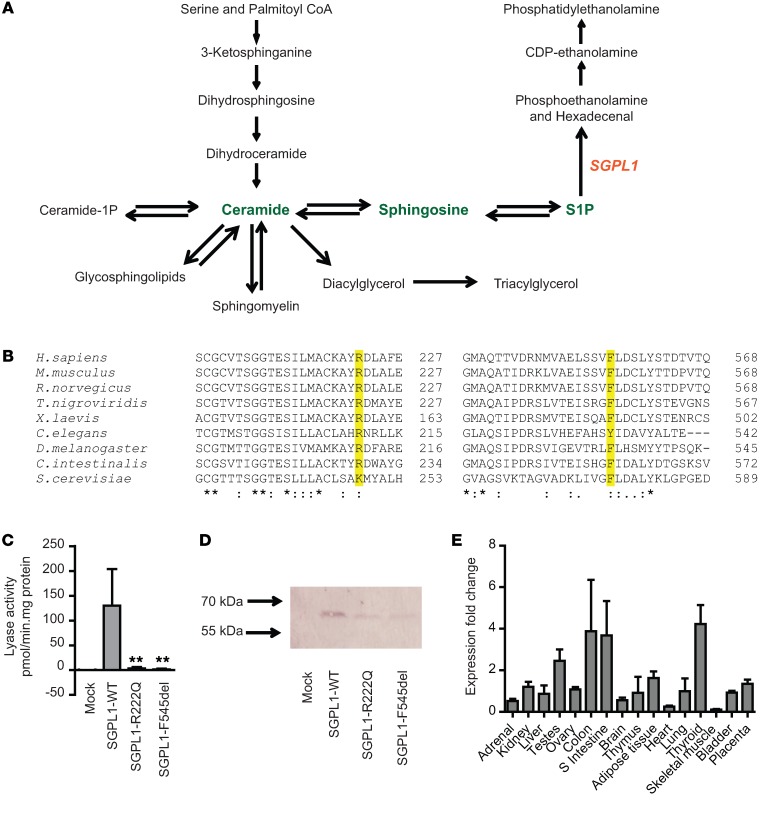
p.R222Q and p.F545del mutations affect highly conserved areas in *SGPL1* and are loss of function, resulting in proteins with reduced lyase activity. (**A**) SGPL1 regulates flow of the sphingolipid biochemical intermediates (in green) and carries out the final degradation step in the pathway. (**B**) Partial alignment of SGPL1 protein sequences, generated by Clustal Omega (48), showing conservation of arginine (R) at position 222 and phenylalanine (F) at position 545, highlighted in yellow, with numbering relative to human sequence. For all but the most distant organisms, these amino acids are conserved. Alignment source accession numbers from ENSEMBL are as follows: *Homo sapiens*, human, ENSP00000362298; *Mus musculus*, mouse, ENSMUSP00000112975; *Rattus norvegicus*, rat, ENSRNOP00000070983; *Tetraodon nigroviridis*, pufferfish, ENSTNIP00000016065; *Xenopus laevis*, clawed frog, ENSXETP00000017960; *Ciona intestinalis*, sea squirt, ENSCINP00000002369; *Drosophila melanogaster*, fruit fly, FBpp0086158; *Caenorhabditis elegans*, nematode, B0222.4; and *Saccharomyces cerevisiae*, yeast, YDR294C. Sequence conservation is beneath the alignment. Asterisks indicate total conservation; colons indicate partial conservation. (**C**) SGPL1 activities were measured in lysates of *Sgpl1^–/–^* mouse fibroblasts. **P < 0.01, 2-tailed Student’s *t* test (*n* = 3). (**D**) Lysates of *Sgpl1^–/–^* mouse fibroblasts expressing WT or the mutant SGPL1 (25 μg protein/lane) were analyzed by immunoblotting for the presence of the FLAG-tagged protein (representative image, *n* = 3). (E) *SGPL1* mRNA expression in a human tissue panel, analysis using the 2^–ΔΔCT^ algorithm (*n* = 3). S intestine, small intestine.

*SGPL1* encodes sphingosine-1-phosphate lyase (SGPL1), an important ER enzyme in sphingolipid catabolism (reviewed in refs. 10, 11). SGPL1 executes the final decisive step of the sphingolipid breakdown pathway, initiating irreversible cleavage of the lipid-signaling molecule sphingosine-1-phosphate (S1P) (Figure 2A).

We subsequently identified further mutations in *SGPL1* in 5 affected patients with a background of PAI from 4 additional families using whole-exome or Sanger sequencing (Figure 1, kindreds 2–5; Table 1). In 4 out of these 5 patients, PAI was also associated with a steroid-resistant nephropathy. In all cases, patients were homozygous for the change and unaffected parents were heterozygous. The c.665G>A (p.R222Q) mutation seen in kindred 1 was also identified in patient 4 (kindred 2), who presented with PAI at 18 months. Interestingly, the R222Q mutations in kindreds 1 and 2 have arisen independently; 2 synonymous exonic (rs827249 and rs865832) and 2 intronic (rs41315008 and rs923177) SNPs, within *SGPL1* itself and surrounding the mutation, differed between patients in these kindreds (Supplemental Table 1; supplemental material available online with this article; doi:10.1172/JCI90171DS1). In kindred 3, a female (patient 5) presented with PAI at 6 months, developing nephrotic syndrome on follow-up at age 5.5 years. Patient 5 and her parents underwent WES and analysis through the GOSgene pipeline (12). Under a homozygous recessive model, the only rare variant that segregated with the disease was an in-frame deletion, c.1633_1635delTTC in *SGPL1*. Kindred 4 comprised 2 affected siblings (patients 6 and 7), manifesting PAI and nephrotic syndrome (< 1 year for both), who had a canonical splice site change (c.261+1G>A; p.S65Rfs*6). Patient 8, presenting with nephropathy (<1 year) and later developing PAI, had a frameshift mutation (c.7dupA; p.S3Kfs*11). We believe that the mutations in kindreds 3, 4, and 5 were novel and have not been annotated in any database. Concurrently, exome data from a cohort of 200 pediatric patients presenting with FSGS alone was examined for S*GPL1* mutations, but none were identified. The frameshift and splice site mutations, p.S3Kfs*11 and p.S65Rfs*6, respectively, are predicted to result in markedly truncated mRNA message and are likely to be destroyed by nonsense-mediated mRNA decay, suggesting that the phenotype results from loss of enzyme activity and may represent a new defect of sphingolipid metabolism. The mutations p.R222Q and p.F545del are within highly conserved residues and are predicted to cause disruption to vital, highly conserved eukaryotic protein domains (Figure 2B). To reveal whether the mutants possessed residual lyase activity, the mutated proteins were expressed in *Sgpl1^–/–^* mouse fibroblasts. The activity of both mutants was close to zero (Figure 2C). The mutations also appear to affect expression or possibly stability of the protein, with both the p.R222Q and p.545del detected at the expected size but in lower amounts than the WT on immunoblotting (Figure 2D).

In agreement with previous studies, SGPL1 was ubiquitously expressed in human tissues, with moderate levels in the adrenal cortex and kidneys (Figure 2E), consistent with rat and mouse expression profiles (13–15). High levels were noted in the testes and thyroid (Figure 2E). Mutations in other components of the sphingolipid breakdown pathway lead to harmful accumulation of lysosomal sphingolipid species, which is associated with a spectrum of conditions known as the sphingolipidoses (16). These include Niemann-Pick disease, Gaucher disease, and Fabry disease, among others, which are multisystemic and often progressive. While a renal phenotype is described in some of these conditions (17), adrenal disease has not been reported to date.

### Human clinical findings.

In our patients, PAI was common to all affected individuals presenting in infancy, with the exception of patient 8, who presented at age 9 years. For most, this was an isolated glucocorticoid deficiency necessitating hydrocortisone replacement; 2 patients additionally had mineralocorticoid deficiency (Table 1). The only postpubertal patient within the cohort, patient 8, also had marked adrenal androgen deficiency (serum dehydroepiandrosterone sulphate level <3 mcg/dl [normal range (NR) 26–460]; androstenedione <1 ng/ml [NR 0.5–4.7]). Adrenal imaging was undertaken in some of the patients. Patient 5 had normal appearance of the adrenals on ultrasound and MRI (undertaken at ages 0.5 years and 5.9 years, respectively). Patient 6 and patient 7 were both reported to have imaging suggestive of calcifications in the adrenals (imaging undertaken at age 1 year and 0.6 years, respectively), with patient 6 additionally reported to have bilateral enlarged adrenal glands.

The nephrotic syndrome was steroid resistant and was present in 5 patients and manifested between the ages of 1.5 months and 5.5 years (Figure 1 and Table 1). Biopsy findings were consistent with FSGS in all affected patients (Supplemental Table 2; further details for patients 1 and 5–7). Electron microscopy of the renal biopsy from patient 5 showed cellular vacuolizations and partial effacement of podocytes (Supplemental Figure 1; electron microscopy of renal biopsy for patient 5). Patients 1, 6, and 8 were post–renal transplant, while patients 5 and 7 were awaiting transplantation at the time of this writing. Patient 8, age 17.5 years, redeveloped proteinuria at 12 years following her initial transplantation at 5 years, necessitating a second renal transplant, a graft biopsy having shown graft failure from chronic rejection.

Extraadrenal and -renal effects are described within our cohort. Generalized ichthyosis was present in most, though not all, patients (Table 1 and Supplemental Figure 2). Skin biopsies conducted in patients 6 and 7 demonstrated a thinned epidermis with hyperkeratosis and decreased granular layer. Primary hypothyroidism was reported in 4 of the patients requiring treatment with l-thyroxine (patients 5–8). Thyroid peroxidase antibodies were negative in all cases.

Neurodegenerative disease is seen in several of the sphingolipidoses associated with accumulating sphingolipid metabolites. Here, progressive motor and cognitive decline is described for 3 patients (patients 5, 6, and 7), with ataxia and sensorineural hearing loss also reported (patients 5 and 6) (Table 1). Both Peruvian siblings (patients 6 and 7) initially presented with development appropriate for age. However, during follow-up, impaired acquisition of new skills was reported. Patient 6 had loss of speech and developed progressive hypotonia and truncal ataxia; at age 8.4 years, this patient was no longer walking. Following development of ataxia at 8 years, cranial MRI showed contrast enhancement of cerebellar structures among other features. Patient 5 was reported to have had normal development as assessed by the Denver II developmental screening test (18) at age 2.3 years. However, assessment at age 4.3 years showed delayed gross motor, language, and social skills. Aged 6 years at the time of this writing, she has developed mild gait abnormalities associated with ataxia. Serial cranial MRI highlighted the progressive nature of her disease (Supplemental Figure 3); at 4.3 years, contrast enhancement was seen in the bilateral globus pallidus, medial thalamic nucleus, and central pons (not observed in a previous scan at 8 months). Ophthalmological findings in this patient included “salt and pepper” retinopathy, cranial nerve III to IV synkinesia, ptosis, and esotropia affecting the right eye. She had complex partial seizures. None of the above-mentioned patients were reported to have autonomic dysfunction. The oldest patient in this cohort (patient 8 at 17.5 years), however, had no intellectual disability and normal neurological findings, including a normal brain MRI (conducted at 11 years).

Fasting lipid profiling revealed raised total cholesterol and triglycerides in some of the patients (Table 1), though in all cases, sampling was conducted with diagnosis of nephrotic syndrome. Mass spectrometric analysis of serum from patient 5 (with p.F545del) revealed increased S1P and ceramide species in comparison with an age- and sex-matched control (Supplemental Figure 4), which would fit with our hypothesis that this disease alters sphingolipid metabolism.

Persistent lymphopenia was a reported feature in 2 of our patients (Table 1). This has been most extensively investigated in patient 5, with lymphocyte subset analysis revealing low CD3^+^ and low naive CD4^+^ and naive CD8^+^ cells, but normal proliferative capacity (Supplemental Tables 3 and 4). Neither of these patients had an increased frequency of infections.

Undervirilization was reported in patient 6, who had micropenis, right cryptorchidism, and bilateral microorchidism, associated with low serum anti-Müllerian hormone, suggesting that partial gonadal dysfunction may be part of the clinical picture.

### Sgpl1^–/–^ mouse adrenal histology and renal histology; expression of SGPL1 in human adrenals.

SGPL1 is highly conserved, with human SGPL1 sharing 84% identity and 92% similarity to mouse SGPL1 (14, 19). The mutations are predicted to be loss-of-function; hence, the *Sgpl1^–/–^* mouse could provide a model for the disorder. We investigated the phenotype of the *Sgpl1*^–/–^ mouse, both our own findings and previous reports in the literature, and compared this with the clinical findings in our patients (Table 2). *Sgpl1*^–/–^ mice are born normally, but fail to thrive at around 2 weeks of age. Most die within the first few weeks of weaning; the reason for this is unknown (20–22). While *Sgpl1^–/–^* mice are reported to have impaired testicular and ovarian steroidogenesis and are infertile (23), an adrenal phenotype has not previously been investigated.

**Figure 3:**
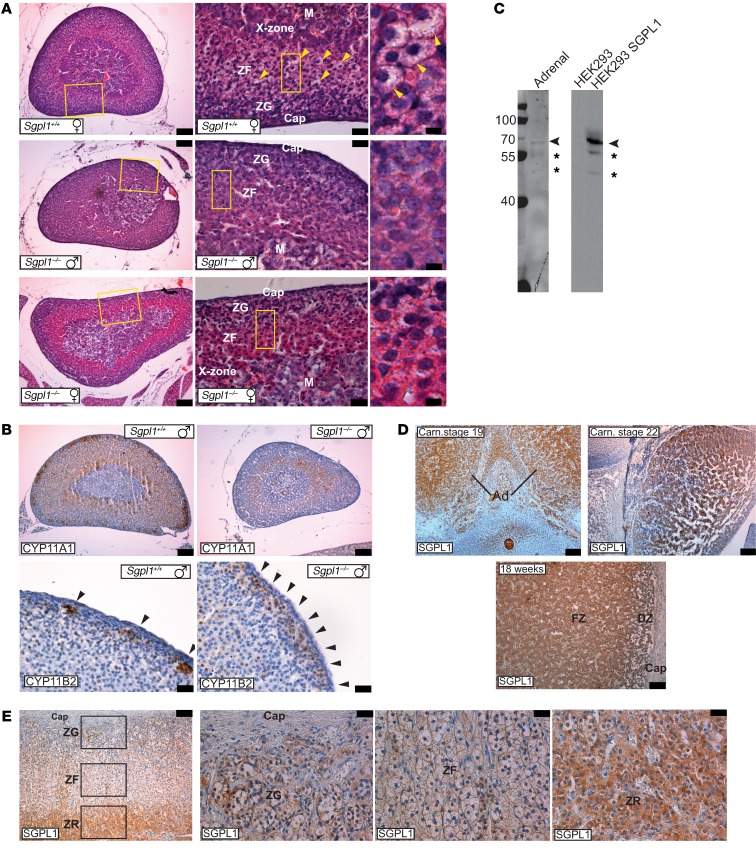
Adrenals from *Sgpl1^–/–^* mice show histological abnormalities, and SGPL1 is expressed in human adrenals. (**A** and **B**) Adrenals from *Sgpl1^–/–^* mice show histological abnormalities. (**A**) H&E staining of *Sgpl1^+/+^* and *Sgpl1^–/–^* adrenals. Note the less defined morphological zonation in the *Sgpl1^–/–^* adrenals compared with that from *Sgpl1^+/+^* mice. Moreover, the characteristic lipid droplets found in the ZF (arrowheads in top-right panel) and visible as large areas in the cytoplasm devoid of eosin staining (as lipids are extracted during the paraffin-embedding procedure) are strongly reduced in *Sgpl1^–/–^* adrenals (*n* = 3). Cap, capsule. Scale bars: 100 μm (left); 25 μm (middle); 5 μm (right). (**B**) CYP11A1 and CYP11B2 expression in *Sgpl1^+/+^* and *Sgpl1^–/–^* adrenals. CYP11A1 staining in *Sgpl1^–/–^* adrenals is less prominent compared with *Sgpl1^+/+^,* while the characteristic patchy expression of aldosterone synthase (CYP11B2) is lost in *Sgpl1^–/–^* adrenals (*n* = 3). Scale bars: 100 μm (top); 25 μm (bottom). (**C**–**E**) Expression of SGPL1 in human adrenals. (**C**) Western blotting of lysates from human adrenal, HEK293, cells and HEK293 cells overexpressing SGPL1 probed with anti-SGPL1 antibody (representative image of *n* = 3). (**D**) SGPL1 expression in the HFA at 19 and 22 Carnegie (Carn) stage as well as at 18 weeks showing widespread expression (*n* = 1 each). FZ, fetal zone; DZ, definitive zone. Scale bars: 100 μm. (**E**) SGPL1 expression in the human adult adrenal (*n* = 3). Note the stronger expression of SGPL1 in the ZR compared with ZG and ZF, while the capsule and medulla (M) are negative. Scale bars: 100 μm (left panel); 25 μm (right 3 panels).

In our study of mouse adrenals, cortical zonation was found to be compromised in 10-day-old *Sgpl1^–/–^* mice regardless of sex, with less definition between zona glomerulosa (ZG) and zona fasciculata (ZF), and between ZF and X-zone (Figure 3A). Cells in the corticosterone-producing ZF were smaller, contained fewer lipid droplets, and had a higher degree of eosinophilia. Compromised development of the X-zone is seen in other mouse models of adrenal insufficiency, including those with *Sf1* mutations (24), suggesting that SGPL1 may also have a role to play in the developing human adrenal. Disruption in steroidogenesis was supported by the analysis of steroidogenic enzymes in the adrenal tissue from these mice. *Sgpl1^–/–^* adrenals showed lower expression of cytochrome P450 side-chain cleavage (CYP11A1), the first enzyme in the steroidogenic cascade, while the classical pattern of subcapsular clusters of aldosterone synthase (CYP11B2) staining present in *Sgpl1^+/+^* mice was replaced by a more continuous pattern in adrenals from *Sgpl1^–/–^* mice (Figure 3B).

Adrenal glands from affected patients were not available for study, but Western blotting confirmed the presence of SGPL1 in normal human adrenals; 3 immunoreactive bands were observed in lysates of adrenals and HEK293-overexpressing SGPL1, indicating the specificity of our antibody in detecting SGPL1 (Figure 3C). SGPL1 was expressed in the human fetal adrenal (HFA) at all stages analyzed, namely Carnegie stage 19 (approximately 46th day) and Carnegie stage 22 (approximately 53rd day) (Figure 3D). At a later stage (18 weeks), when the fetal and definitive zones (which later constitute the ZG, ZF, zona reticularis [ZR]) are distinguishable, SGPL1 was found to be expressed in both (Figure 3D). In the adult human adrenal, SGPL1 immunoreactivity was seen throughout the cortex, with the highest signal in the ZR, responsible for adrenal androgen secretion, and very little expression in the medulla and capsule (Figure 3E).

**Figure 4:**
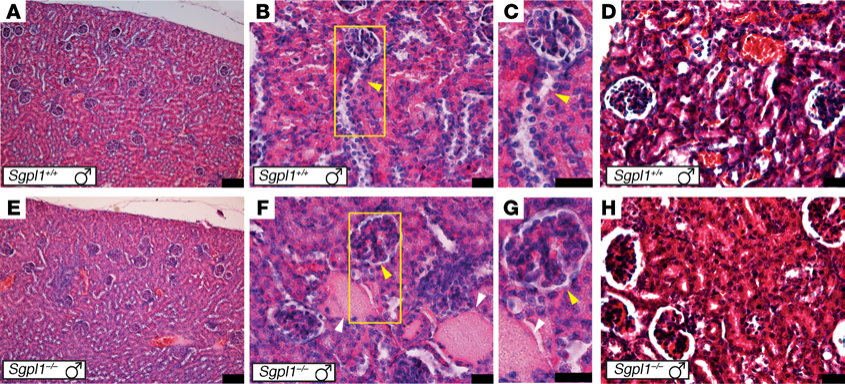
Histological features of the glomeruli. (**A**–**C**) H&E staining of *Sgpl1^+/+^* kidney showing normal cortical histology (**A**) and glomeruli with open capillary loops and normal cellularity (**B** and **C**, yellow arrowhead). The kidneys from *Sgpl1^–/–^* mice (**E**–**G**) have mild mesangial hypercellularity with glomerular hypertrophy (**F** and **G**, yellow arrowhead) and large protein casts in the tubules (white arrows). (**D** and **H**) Masson’s trichrome stain. Kidneys from *Sgpl1^–/–^* mice (**H**) show increased glomerular fibrosis (red stain) compared with *Sgpl1^+/+^* (**D**). *n* = 3 in all cases. Scale bars: 100 μm (**A** and **E**); 25 μm (**B**–**D** and **F**–**H**).

Consistent with previous reported findings in SGPL1-deficient mice (21, 25), we saw mesangial hypercellularity and proteinaceous casts in the tubules of *Sgpl1*^–/–^ mice, with overall histological appearances supporting a glomerular phenotype (Figure 4, A–H). Moreover, a higher degree of glomerular fibrosis was observed in kidneys from *Sgpl1*^–/–^ mice using Masson’s trichrome stain (Figure 4, D and H).

## Discussion

This is the first instance of SGPL1 deficiency in humans highlighting a novel biochemical player involved in adrenal disease. The sphingolipids form one of the major classes of the mammalian lipidome. They have multiple roles: some as structural components of cell membranes (sphingomyelin, glycosphingolipid) and others as signaling molecules (ceramide, sphingosine, S1P). S1P has autocrine, paracrine, and distant effects, regulating cell migration, differentiation, survival, and other complex physiological processes by activating a subgroup of G protein–coupled receptors referred to as S1P_1–5_. SGPL1, an intracellular ER enzyme, carries out the final decisive step of the pathway with degradation of intracellular S1P, regulating flow of the sphingolipid biochemical intermediates, and therefore has a key role in cell metabolism and determining cell fate (Figure 2A).

Lipids, including sphingolipids, have been shown to control steroid hormone biosynthesis in adrenal glands. Sphingolipid intermediates ceramide and sphingosine have been demonstrated to reduce steroidogenesis in vitro (26–28). Sphingosine has been reported to interact directly with the orphan nuclear receptor steroidogenic factor-1 (SF-1; NR5A1), which plays a role in both the acute phase of steroidogenesis and in adrenal and gonadal development (28). In binding to SF-1, sphingosine maintains the transcription factor in an inactive conformation. The sphingosine intermediate S1P, however, has been demonstrated to induce transcription of several steroidogenic factors (29). Thus, sphingolipid intermediates may have some role in modulating steroidogenesis and potentially adrenal development. We saw increased plasma S1P and ceramide levels in patient 5, and we anticipate that SGPL1 deficiency would lead to cytosolic accumulation of sphingolipid intermediates. Studies of tissues from SGPL1-deficient mice demonstrate an accumulation of S1P (22, 25, 30). While S1P is largely considered a proproliferative signaling molecule and ceramide proapoptotic in normal physiology, studies have demonstrated that pathological accumulation of S1P in the cytosol can induce apoptosis (31).

In contrast with what occurs in the mouse, SGPL1 deficiency is not lethal in humans, but in many aspects, the mouse phenotype mirrors the human condition. In the mouse, imperfect adrenal gland zonation together with a reduction in expression of steroidogenic enzymes and loss of vacuolization in the ZF are consistent with the biochemical finding of adrenal hormone insufficiency in patients. Significant SGPL1 expression is observed in the kidney by analysis of β-galactosidase expression in organs dissected from male and female SGPL1 reporter mice, with specific patterns of expression in the cortex, medulla, and papilla (13). Images from The Human Protein Atlas (http://www.proteinatlas.org/) show SGPL1 expression in the tubules and glomeruli in normal human adult kidney; within the glomerulus, staining is apparently predominantly in podocytes (see Supplemental Figure 5). *Sgpl1*^–/–^ mice have previously been reported to have renal defects, with increased blood urea levels (21), and a renal phenotype was confirmed in our studies, with histological changes in the kidney matching the human biopsy results. Inducible SGPL1 deficiency in mice and pharmacological inhibition of SGPL1 in rats both cause podocyte-based kidney toxicity, with glomerular proteinuria (25). Increased mesangial matrix with obliteration of some capillary lumina is seen in the SGPL1-deficient mouse model with podocyte effacement on electron microscopy (25).

Additionally, ichthyosis, disordered lipid metabolism, and lymphodepletion are shared in some patients with the mouse, whereas the neurological deficits and hypothyroidism have not been noted in mice. It is feasible that some of the clinical findings in patients are attributable to alternative gene defects, given the high degree of consanguinity within the pedigrees.

Ceramide species constitute 50% of the lamellar membrane of the stratum corneum and play an important role in maintaining skin barrier function (32). Ichthyosis is also seen in Gaucher disease type 2, where substantial reductions in lysosomal β-glucocerebrosidase lead to excess glucosylceramides and decreased ceramides, imbalance of which is postulated to contribute to the barrier abnormality (33). In the skin of partially depleted SGPL1 mice, a rise in S1P concentrations is seen with a reduction in ceramide concentrations that may in turn interfere with barrier function (25). The phenotype in these mice is one of acanthosis (epidermal hyperplasia) with orthokeratotic hyperkeratosis.

We observed high levels of SGPL1 in human thyroid tissue (Figure 2E) and 4 of the patients had primary hypothyroidism; however, mild disruptions of thyroid function are a reported phenomenon with PAI, particularly in the context of untreated disease (34–36). Additionally, proteinuria, particularly in nephrotic syndrome, often results in the urinary loss of thyroid hormones with associated binding proteins, resulting in a reduction in serum total thyroid hormone levels (37). This is generally equilibrated by increasing the free fraction of the hormones, though patients with a low thyroid reserve may develop hypothyroidism consequent to this urinary loss.

Progressive neurological disease was reported in 3 of our patients, but has not been reported in *Sgpl1^–/–^* mice. Curiously, compared with other organs, SGPL1 expression is low in rat and mouse brain (13, 15). Within the brain, SGPL1 shows a distinct expression pattern, with highest activity in the olfactory bulb in mouse (15, 38). Other regions with higher expression are the hippocampus (P.P. Van Veldhoven, unpublished observations) and Purkinje cells in the cerebellum (13, 31). Neuroanatomical studies in the cerebellum demonstrate that neurons here are the first to degenerate in SGPL1-deficient mice (31). A calpain-mediated neurotoxic mechanism has been proposed relating to pathological accumulation of cytosolic S1P in SGPL1 deficiency (31).

*Sgpl1*^–/–^ mice exhibit an extensive range of other phenotypes, including skeletal (osteopetrosis and dysfunctional osteoclasts), pulmonary (proteinaceous exudates in alveoli impairing gas exchange), cardiac (increased interstitial cellularity and myocardium vacuolation), urinary tract (urothelial cells affected by widespread ballooning vacuolation, degeneration, and apoptosis), thymic (atrophy), and hepatic abnormalities (disruption of liver homeostasis with defective hepatic lipid metabolism) (22, 30, 39). Hematological abnormalities associated with lyase deficiency in the mouse model include platelet activation and lymphodepletion, which occurs as a consequence of the disruption of the S1P gradient between lymphoid tissues and blood (normally high to low, respectively). This leads to reduced T cell egress into the blood (22, 39). Similarly, platelet abnormalities and lymphopenia were reported in some of our patients (Table 1). Inhibition of SGPL1 in healthy subjects has also been demonstrated to cause a decrease in peripheral T cell numbers (22). Partial restoration of S1P lyase in human knockin mouse lines (expressing 1 or 2 normal human alleles and less than 10% and 20% of normal lyase activity, respectively) confers full protection from lesions in the lung, bone, urinary tract, and heart that develop in SGPL1 null mice (22). Lymphodepletion, however, still occurs in the partially rescued human knockin mouse line.

In conclusion, we have identified a potentially progressive, disorder incorporating PAI and nephrotic syndrome, among other features. SGPL1 deficiency in humans has multisystemic effects, although the precise impact on sphingolipid metabolism and consequent pathogenic mechanism is yet to be elucidated and is likely to be tissue specific. Our findings highlight the importance of the sphingolipid metabolic pathway in adrenal function. A genetic diagnosis for patients with this form of PAI will be important for correct treatment, genetic counseling, and screening for comorbidities.

Since strategies to modify SGPL1 activity are being explored in preclinical and clinical trials of several conditions, including multiple sclerosis and rheumatoid arthritis (40, 41), further characterization of SGPL1 deficiency in human disease may provide important insights into the potential long-term effects of modulating this pathway in patients.

## Methods

### WES.

WES using the Illumina HiSeq 2000 Sequencer was conducted on 2 affected individuals (patients 2 and 3) (samples processed by Otogenetics Corp.). WES samples were prepared as an Illumina sequencing library, and in the second step, the sequencing libraries were enriched using the Agilent V4 Enrichment Kit. The captured libraries were sequenced and downstream analysis conducted via DNAnexus (https://www.dnanexus.com/) and Ingenuity variant analysis. SNPs with threshold coverage of at least 10 reads on the respective nucleotides were assessed.

### Confidence filter.

For confidence, call quality was set to be at least 20, read depth at least 10 in any sample; only data outside 0.1% of most exonically variable 100-base windows in healthy public genomes and outside 0.1% of most exonically variable genes in healthy public genomes (1000 Genomes, http://www.internationalgenome.org/, EXAC) were included.

### Common variants.

Common variants were filtered out by excluding those observed with an allele frequency of at least 0.5% in any of the 1000 Genomes, EXAC, and all of the NHLBI exomes.

### Predicted deleterious changes.

Predicted deleterious changes were defined as those that are disease associated according to computed American College of Medical Genetics and Genomics (ACMG) guidelines classification (42), being pathogenic or likely pathogenic or associated with loss of function of a gene being frameshift, in-frame indel, or start/stop codon change, missense, or splice-site change up to 2 bases into the intron.

### Genetic analysis.

Variants were included if they were homozygous in both cases and excluded if they were homozygous, compound heterozygous, hemizygous, heterozygous, haploinsufficient, or het-ambiguous. Variants were excluded if they were present in hetero- or homozygosity in 2 or more control subjects analyzed on the same platform.

### PCR and sequencing.

Each exon of genes of interest, including intronic boundaries, was amplified by PCR using specific primers (primer sequences, Supplemental Table 5). The reaction mixture contained 100 ng DNA template, 1× PCR buffer, 200 μM each dNTP, 200 nM each primer, and 1 U *Taq* DNA polymerase (Sigma-Aldrich). Cycling conditions were as follows: 95˚C for 5 minutes (1 cycle); 95˚C for 30 seconds, 55˚C for 30 seconds, and 72˚C for 30 seconds (30 cycles); and 72˚C for 5 minutes. PCR products were visualized on 1% agarose gel and sequenced using the ABI Prism Big Dye Sequencing Kit and an ABI 3700 automated DNA sequencer (Applied Biosystems) in accordance with the manufacturer’s instructions.

### Quantitative real-time PCR.

The expression of *SGPL1* and *GAPDH* mRNA was investigated using a panel of cDNAs derived from 16 adult tissues (adrenal cortex, kidney, liver, testes, ovary, colon, small intestine, brain, thymus, adipose tissue, heart, lung, thyroid, skeletal muscle, bladder, placenta).

Quantitative reverse-transcriptase PCR (RT-PCR) was set up in duplicate (per sample) on a Stratagene Mx3000P thermocycler using KAPA SYBR Fast qPCR Master Mix with 200 nM forward and reverse primers targeted to *SGPL1* or *GAPDH* (primer sequences, Supplemental Table 5), giving a total volume of 10 μl. After an initial denaturation step of 3 minutes at 95°C, PCR cycling was performed for 40 cycles of 95°C for 3 seconds, 55°C for 20 seconds, and 72°C for 1 second, followed by 1 cycle of 1 minute at 95°C, 55°C for 30 seconds, and 95°C for 30 seconds. The 2^–DDCT^ algorithm was used for analysis, with normalization to *GAPDH*.

### Vector construction: site-directed mutagenesis.

A commercially available SGPL1-human (Myc-DDK–tagged) SGPL1 expression vector (TruOrf Gold; Cambridge Bioscience, catalog RC208705) was used. Site-directed mutagenesis was carried out to prepare the R222Q and F545del constructs (primer sequences, Supplemental Table 6) using the QuikChange II Site-Directed Mutagenesis Kit (Agilent) per the manufacturer’s instructions. The sequence of all constructs was verified by DNA sequencing.

### Lyase activity studies.

Mouse fibroblasts lacking SGPL1, obtained from *Sgpl1^–/–^* embryos (20) and immortalized by dilution culturing (43), were cultured in MEM-Eagle medium with 10% FBS, 2 mM ultraglutamine-1, and 0.2% Mycozap. Cells were transfected with SGPL1 vectors by electroporation (Neon Transfection System) using the parameters described for SV40-transformed MEF and a 10-μl microporator tip (44). Per plasmid tested, 3 aliquots of 10 μl transfected cells (~5 × 10^5^ cells) were seeded in a 90-mm dish. Two days after transfection, cells were collected by trypsinisation, centrifuged, and washed with PBS. The pelleted cells, stored at –20°C till use, were adjusted to approximately 250 μl, with 0.25 M sucrose, 5 mM MOPS, pH 7.2, 1 mM EDTA, and 0.1% (v/v) ethanol, and sonicated (Hielscher Ultrasonic tissue homogenizer UP50H; 3 pulses of 50 W) on ice. Lyase was measured as described before (45), with small modifications. The activity represents the amount of hexadecanal formed from sphinganine-1-phosphate and is corrected for endogenous levels; values are given in pmol substrate converted per minute per mg lysate protein (pmol/min/mg protein).

### Western blotting.

HEK-293 cells were transfected with the SGPL1 vector using Lipofectamine 2000 (Invitrogen) according to the manufacturer’s instructions. After 48 hours, cells were lysed in RIPA buffer (Sigma-Aldrich) supplemented with complete protease inhibitors (Roche) and lysates cleared by centrifugation at 13,000 *g* for 10 minutes at 4 °C; supernatants were added to an equal volume of reducing Laemmli loading buffer 2× (Sigma-Aldrich). Normal adult adrenals (*n* = 3) were placed in tubes with RIPA buffer supplemented with complete protease inhibitors and lysates obtained using a Precellys24 Tissue Homogenizer via bead beating technology. Lysates were then cleared and prepared as above. Samples were heated at 95–100˚C for 5 minutes and size-separated on 4%–12% SDS-PAGE gels (NuPage, Thermo Scientific). Proteins were then transferred to nitrocellulose membrane (GE Healthcare Life Sciences) using a semi-dry transfer blot (Bio-Rad) at 15 V for 1 hour. Blots were blocked with 5% nonfat dry milk in PBS–Tween 0.1% (blocking buffer) and immunolabeled overnight with a polyclonal anti-SGPL1 antibody (105183, Abcam, at 1:1000 in blocking buffer). The following day, the membrane was washed 3 times in PBS–Tween 0.1% and then incubated with a goat anti-rabbit IRDye 800 (926–6871, LI-COR) at a 1:5,000 dilution, washed again, and visualized using the LI-COR Odyssey System.

For visualization of SGPL1 in transfected mouse fibroblasts, lysates were size separated as above and membranes incubated with anti-FLAG antibodies (200472-21, Roche), followed by alkaline phosphatase–conjugated secondary antibody (A2429, Sigma-Aldrich). NBT/BCIP (Roche) was used for chromogenic detection.

### Generation and validation of mouse lines.

Heterozygous *Sgpl1^–/+^* animals, obtained as described before (20, 43, 46) and bred to a C57BL/6 background, were crossed to obtain *Sgpl1^–/–^* pups. Animals were housed in the animal facility of KU Leuven.

### Human and mouse histology.

Human adult adrenals, mouse adrenals, and kidneys from *Sgpl1^+/+^* and *Sgpl1^–/–^* mice were fixed in 4% paraformaldehyde (Sigma-Aldrich) and embedded in paraffin. HFAs were obtained from the MRC–Wellcome Human Development Biology Resources (HDBR) – Institute of Genetics Medicine, Newcastle, United Kingdom). Sections were obtained using a microtome (Microm HM 325, Thermo Fisher) at 6-μm thickness, deparaffinized, treated with 3% hydrogen peroxide in PBS for 30 minutes, and then, depending on the primary antibody (see below), treated or not with 10 mM sodium citrate, pH 6.0, for 20 minutes at 95^o^C in a water bath. Sections were then blocked with 5% normal goat serum (Sigma-Aldrich) in PBS 0.1% Triton X-100 (T-PBS) and incubated overnight with anti-CYP11B2 (a gift from Celso Gomez-Sanchez, University of Mississippi, Oxford, Mississippi, USA, 1:200 in PBS, with previous antigen unmasking), anti-CP11A1 (D8F4F, Cell Signaling, 1:200 in PBS, without previous antigen unmasking), and anti-SGPL1 (105183, AbCam, 1:200 in PBS, without previous antigen unmasking). Sections were washed in T-PBS and incubated with a biotinylated goat anti-primary IgG secondary antibody (BA-9200 and BA-1000,Vector Laboratories) diluted 1:500 in T-PBS for 2 hours. Sections were washed, treated with avidin-biotin complex (ABC Elite Kit, Vector Laboratories) according to the manufacturer’s instructions, rinsed again, and incubated with the ImmPACT DAB peroxidase substrate kit (Vector Laboratories). The reaction was stopped with H_2_O, and slides were counterstained with hematoxylin, dehydrated, and coverslipped using Vecta Mount (Vector Laboratories).

H&E staining was performed using standard procedures. For fibrotic changes in the kidney, Masson’s trichrome staining kit (Sigma-Aldrich) was employed. Images were acquired using a Leica DM5500B microscope equipped with a DCF295 camera (Leica) and DCViewer software (Leica), and then processed with Adobe Photoshop CS6 and Adobe Illustrator CS6.

### Statistics.

In studies in which statistical analyses were performed, a 2-tailed Student’s *t* test was used to generate *P* values. *P* values less than or equal to 0.05 were considered significant. Data are presented as mean ± SD in all figure parts in which error bars are shown.

### Study approval.

This study was approved by the local hospital ethics committees, and all parents (and children, when possible) gave written informed consent. Approval was obtained from the Outer North East London Research Ethics Committee, reference number 09/H0701/12, the Marmara University Ethics Committee, reference number B.30.2.MAR.0.01.02/AEK/108, and the Vall d’Hebron Hospital Ethics Committee (Paediatric Endocrinology Collection at Vall d’Hebron Biobank). All experiments involving mice were approved by the Ethical Committee for Animal Experimentation (ECD) of KU Leuven (project P196/2012, granted to PPVV; laboratory license number LA1210260).

### Note added in proof.

Since the submission of this paper, *SGPL1* deficiency has been described as the cause of Charcot-Marie-Tooth neuropathy in 2 siblings (47).

## Author contributions

LAM and RP designed the study. NR, TG, DB, IB, EB, HC, MFC, NC, F-C, UD, RK, BH, ES, EY, RP, and HLS recruited and clinically characterized patients. RP, LAM, FB, JCA, TG, and AB analyzed WES data. RP, AM, and LAM conducted Sanger sequencing and analysis of data. AM, EM, and RP generated mutant constructs, and PPVV conducted lyase activity experiments. PPVV generated and validated mouse lines. IH, LG, JH, and MS conducted mouse/human adrenal and kidney histology. RP and LAM prepared the draft manuscript. All authors contributed to the discussion of results and edited and approved the final manuscript.

## Supplementary Material

Supplemental data

## Figures and Tables

**Table 2:**
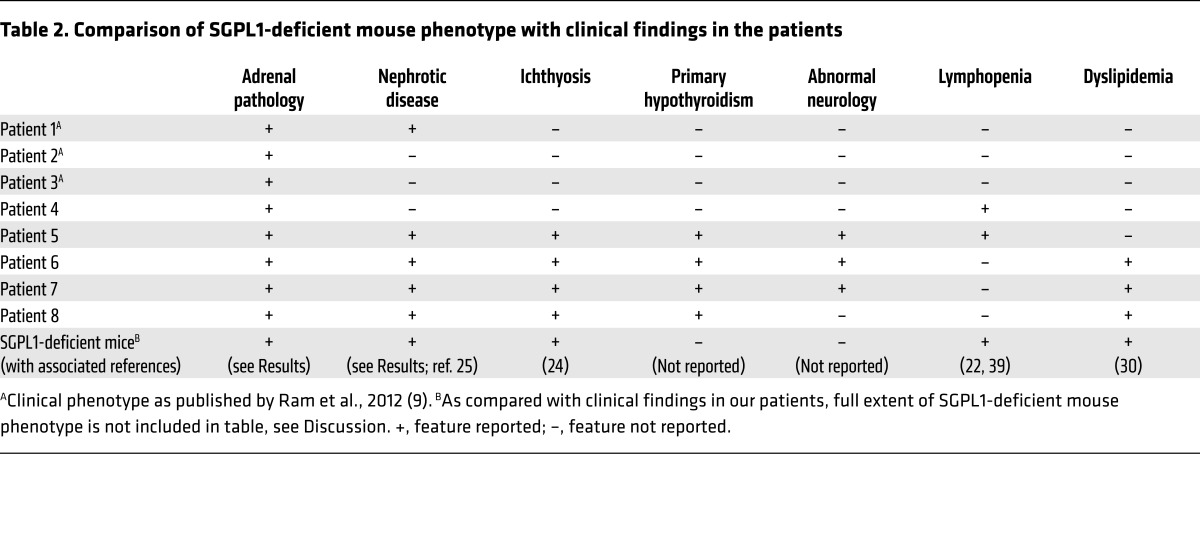
Comparison of SGPL1-deficient mouse phenotype with clinical findings in the patients

**Table 1:**
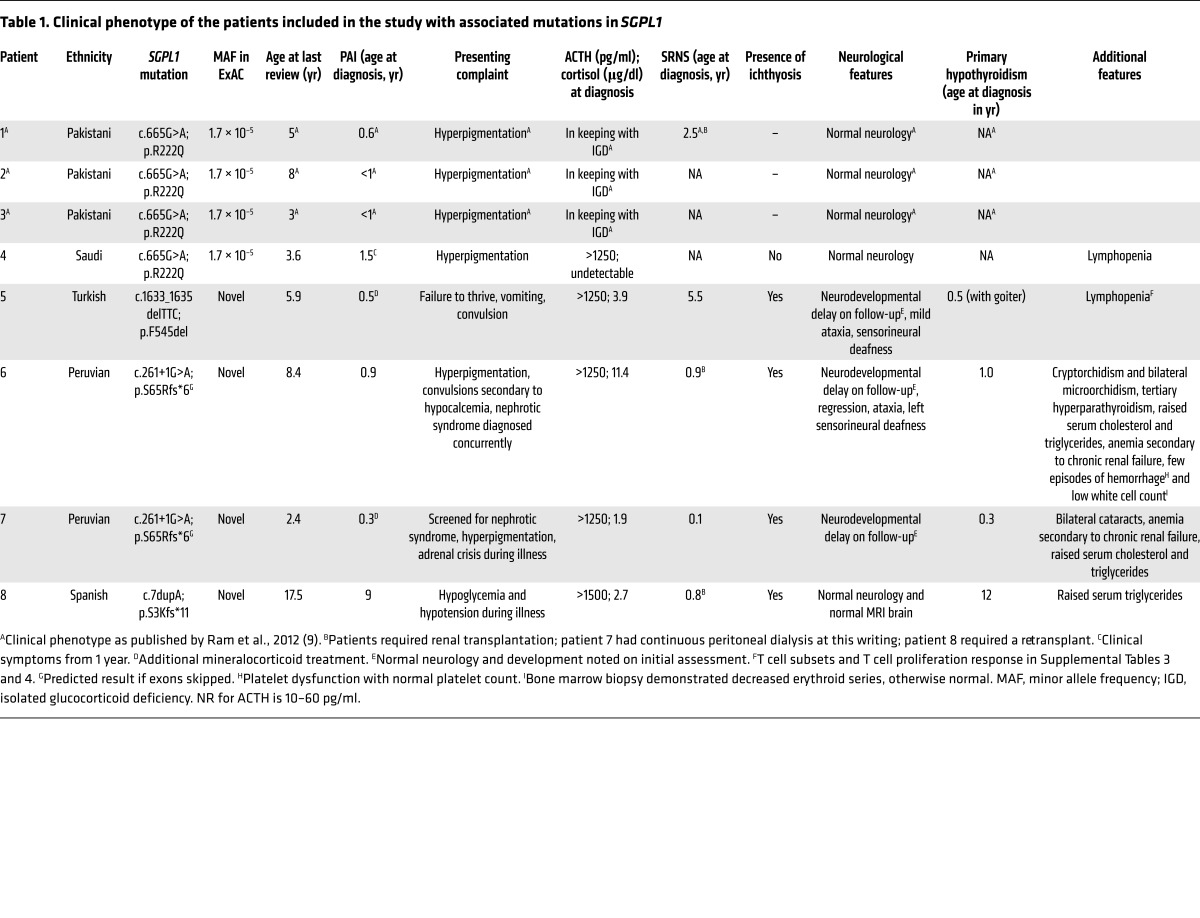
Clinical phenotype of the patients included in the study with associated mutations in *SGPL1*
