# Supplementary material for: Sphingosine-1-phosphate lyase mutations cause primary adrenal insufficiency and steroid-resistant nephrotic syndrome
Source: J Clin Invest. 2017 Feb 6;127(3):942–53. doi: 10.1172/JCI90171 (PMC5330744; doi:10.1172/JCI90171)
Supplement: Supplemental data [file jci-127-90171-s001.pdf]

Supplementary Figures

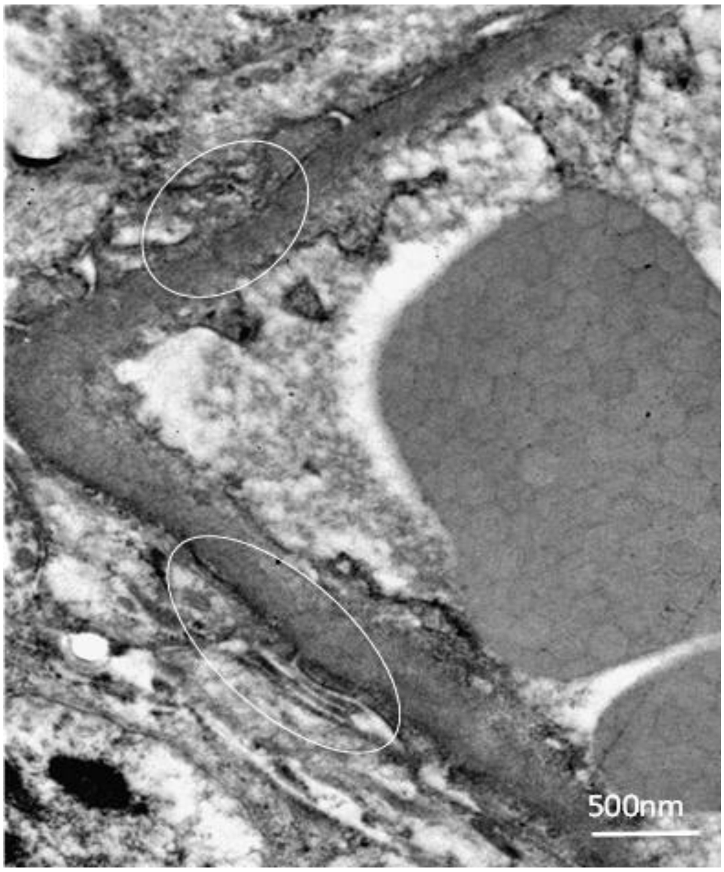

Supplementary Figure 1. Representative image of electron microscopy of renal biopsy from Patient 5, with widespread partial podocyte foot effacement (circled) and flattening of foot processes.

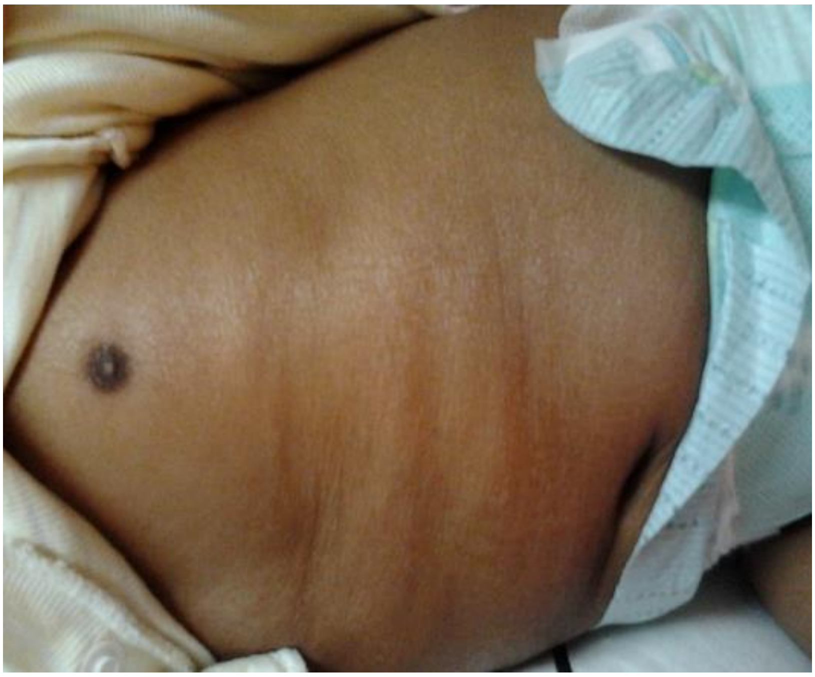

Supplementary Figure 2. Clinical finding of ichthyosis for patient 7, hyperpigmentation as a consequence of primary adrenal insufficiency is also seen

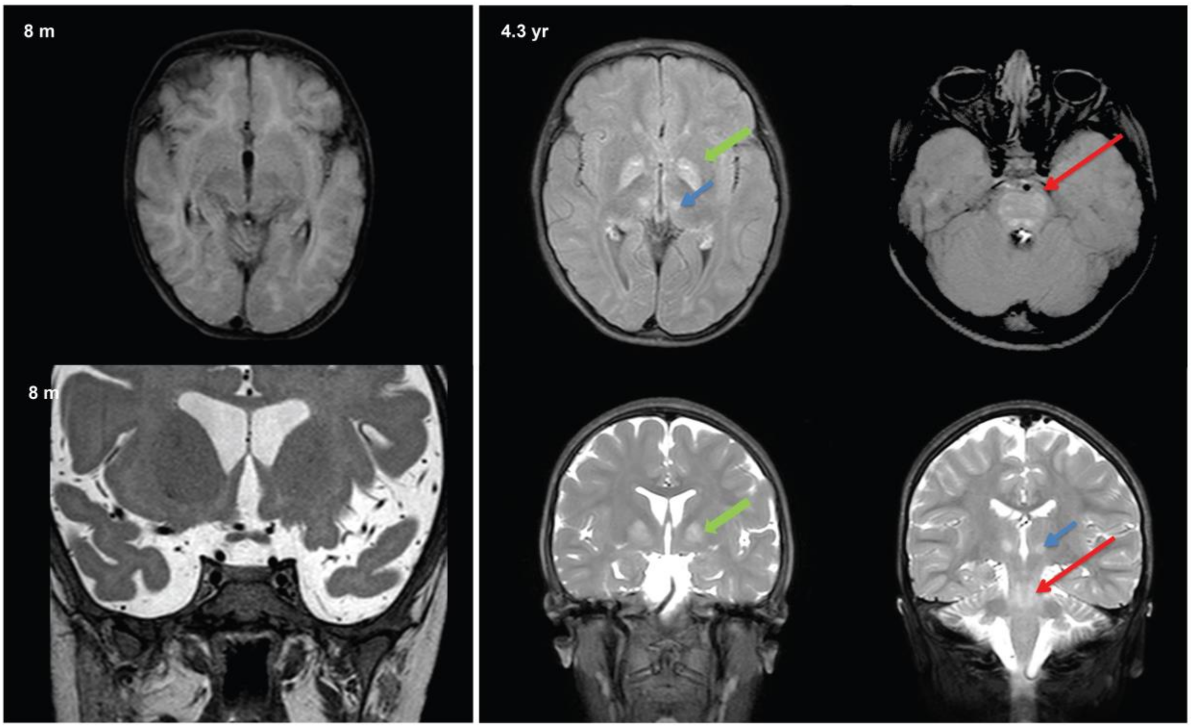

Supplementary Figure 3. Cranial MRI of patient 5 showing the progressive nature of her disease, aged 8m and 4.3yr. At 4.3 yr (right panel), hyperintensity is seen in the globus pallidus (green arrow), medial thalamic nuclei (blue arrow) and pons (red arrow). These features were not seen on scanning at 8 m.

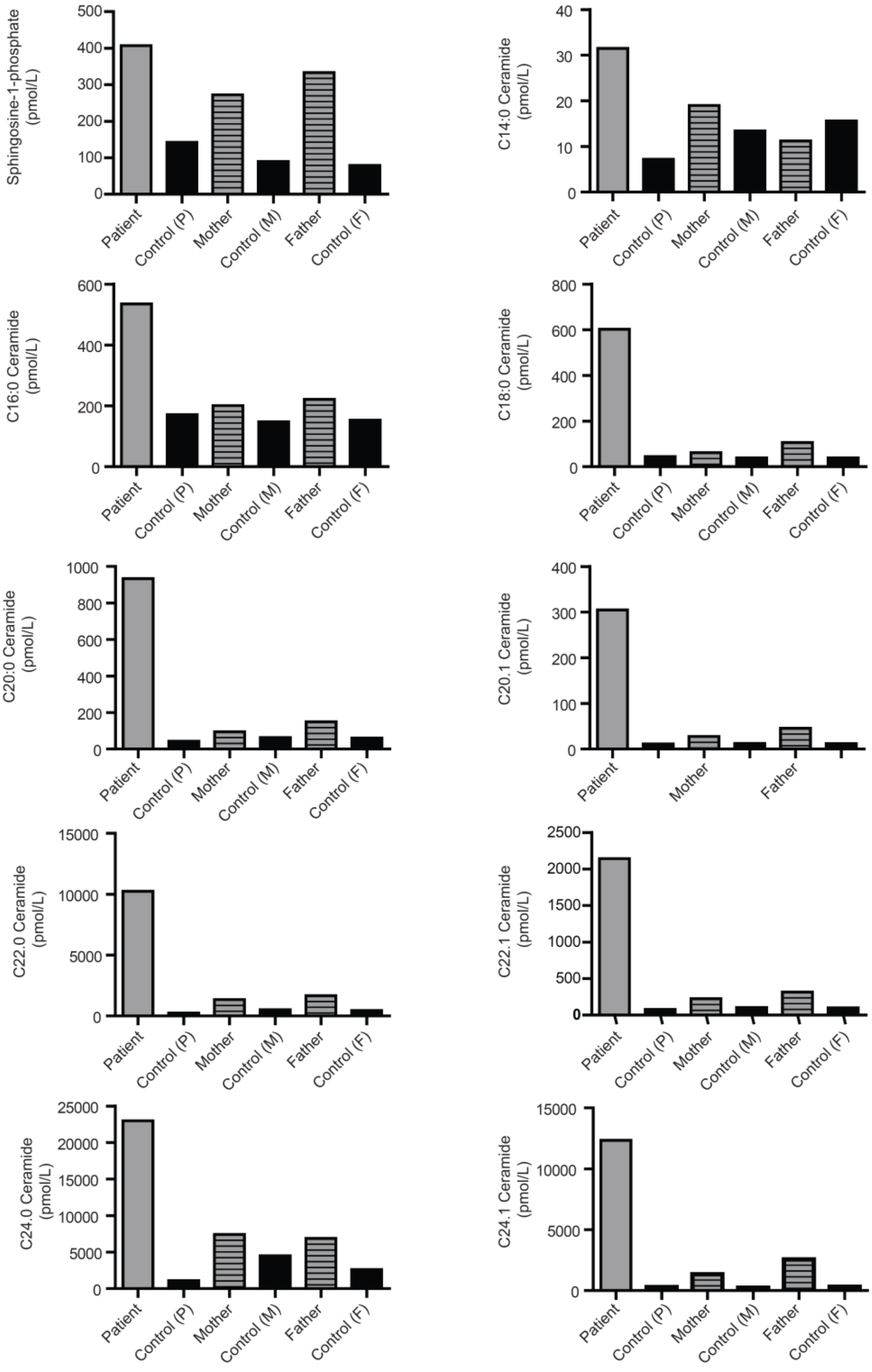

Supplementary Figure 4. Plasma sphingolipid intermediate levels, assayed by mass spectrometry, of Patient 5 and heterozygote parents with age and sex-matched controls

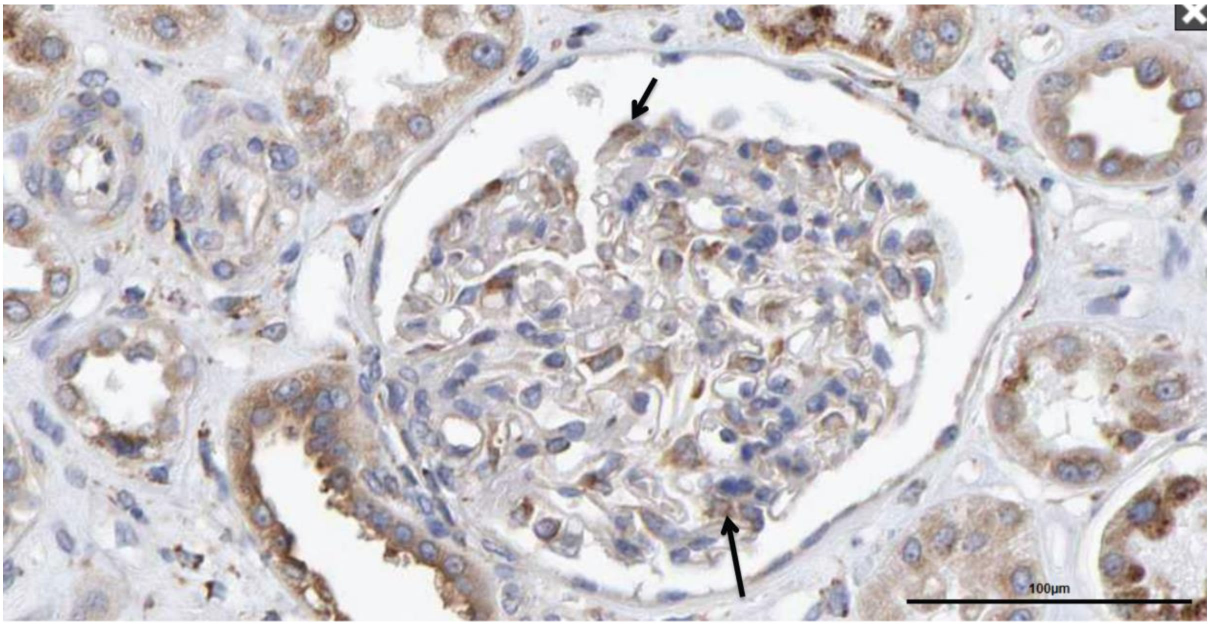

Supplementary Figure 5. SGPL1 expression in the tubules and glomeruli in normal human adult kidney; within the glomerulus staining is apparent predominantly in podocytes (arrowed); Source: Human protein atlas (http://www.proteinatlas.org/) where sections from a normal adult human kidney have been stained using a rabbit polyclonal antibody against SGPL1 (Sigma-Aldrich, HPA023086).

Supplementary Table 1. The mutation resulting in p.R222Q and surrounding SNPs in Kindreds 1 and 2.

Chromosome	Position	Reference Allele	Gene Region	Gene Symbol	Protein Variant	Family 1 allele	Family 2 allele	Translation Impact	SIFT Function Prediction	PolyPhen-2 Function Prediction	dbSNP ID	NHLBI ESP Frequency (%)	NHLBI ESP African Frequency (%)	NHLBI ESP European Frequency (%)	ExAC Frequency (%)
10	72576882	T	Intronic	SGPL1		C	T				41315008				0
10	72619205	C	Exonic	SGPL1	p.I188I	T	T	synonymous			827255	98.25	94.87	99.99	99.55
10	72628151	G	Exonic	SGPL1	p.R222Q	A	A	missense	Damaging	Probably Damaging	769259446	0	0	0	1.70E-05
10	72629567	C	Exonic	SGPL1	p.A241A	C	T	synonymous			827249	3.98	4.27	3.84	5.34
10	72631626	C	Exonic	SGPL1	p.V314V	C	T	synonymous			865832	16.45	9.08	20.22	17.64
10	72636450	T	Intronic	SGPL1		G	T				923177	73.21	73.35	73.14	76.58

Supplementary Table 2. Further details of kidney biopsy results for patients with SRNS

Patient	Biopsy report
1[A]	Light microscopy: Focal segmental glomerulosclerosis<br><br>Immunofluorescence: Granular membrane and mesangial positivity of IgM, weak positivity of C3 and C1q in the same distribution; IgG and IgA negative
5	Light microscopy: Focal segmental glomerulosclerosis, tubular regenerative changes<br><br>Immunofluorescence: Glomerular/ focal/ segmental positivity of IgM, C3 positive, kappa/ λ weakly positive; IgG negative, IgA negative, C19 negative<br><br>Electron microscopy: Vacuolisation of tubular podocytes, podocyte foot process effacement and conglomeration, increased numbers of abnormally shaped mitochondria in tubules (rectangular, comma shaped swollen mitochondria)
6	Light microscopy: 20 glomeruli with more than 10 showing global sclerosis. Increase in mesangial matrix and mesangial hypercellularity. Adhesion the Bowman´s capsule. 2 glomeruli with cellular crescents. Interstitial: Tubular dilatations and tubular atrophy<br><br>Immunofluorescence: IgM positive, IgA negative, IgG negative; C3 positive, C1q negative
7	Light microscopy: Nine glomeruli, one collapse. Increase in mesangial matrix and mesangial hypercellularity. Adhesion the Bowman's capsule. Podocyte hyperplasia and hypertrophy with vacuoles. Interstitial fibrosis and focal tubular atrophy and tubular dilatations.<br><br>Immunofluoresence: negative

[A]As published by Ram and colleagues, 2012 (9)

Supplementary Table 3. Immunophenotyping of Patient 5. Sampling at 5.9 yr, techniques as previously described (1); Peripheral blood lymphocyte numbers: 1000 cells/µl; 19.4% of total leucocyte count (NR 21-40)). TCR αβ; T-cell receptor (TCR) α- and β-chain, TCR Yδ; T-cell receptor (TCR) Y- and δ-chain.

Subset	% Cells	Cells/µl	Normal range (NR)	Comment
$CD3^+$	64.9	640	(>700)	Low
$CD3^+$ $CD4^+$	30.3	300	(>300)	Normal
$CD3^+$ $CD8^+$	27.1	270	(>300)	Low
$CD3^+CD4^-CD8^-$	2.09			Normal
T cell repertoire (TCR αβ)	92			Normal
T cell repertoire (TCR yΔ)	7.5			Normal
$CD3^+$/$HLADR^+$	8.8		(3-14%)	Normal
Recent thymic emigrant (RTE)	61.4		(41-81%)	Normal
Naïve $CD4^+$	29.8	(53-86)		Low
Memory $CD4^+$	43.4	(9-26)		High
Naïve $CD8^+$	60.8	(>69)		Low
Memory $CD8^+$	40	(4-16)		High
$CD19^+$		160	(>200)	Low
$CD16^+$/$CD56^+$		140	(>90)	Normal
Class-switched B cell	12.2		(>10.9%)	Normal
Unclass-switched B cell	20.5		(5.2-20.4%)	Normal

Transitional B cell	1.03		(>4.6%)	Low
Plasmablast	8.3		(0.6-5.3%)	High

Supplementary Table 4. T lymphocyte proliferation assay of Patient 5

	Normal saline stimulation[A] (%)	Phytohaemagluttinin stimulation[B] (%)	CD mix[C] (%)
Patient 5	5	41	66.7
Age and sex matched control	6	23.4	40.4

The ability of T lymphocytes to proliferate in response to [A]saline, a [B]mitogen (Phytohaemagluttinin) or [C]anti–T cell receptor (anti-CD3/CD28 antibody; CD mix) was determined *in vitro*. Relative T cell expansion is compared to an age and sex matched control. Assays performed at 5.9 yr, techniques as previously described (1).

Supplementary Table 5. Primer Sequences

Name	Sequence (5' to 3')
***SGPL1* EX2 for**	AGGAGGGAGAGAACCATAACT
***SGPL1* EX2 rev**	AGCAAGCATCAGAGGTGA
***SGPL1* EX3 for**	GAATGACCTTGCCCTTGA
***SGPL1* EX3 rev**	ACTCCAGCCTAGCAACAGA
***SGPL1* EX4 for**	ACTCTTTGCAATTGGAAGG
***SGPL1* EX4 rev**	CCTCCACTTTGAGAATATTAGGTT
***SGPL1* EX5 for**	AGCAGTTGCTTGACTGTCA
***SGPL1* EX5 rev**	GAAATTCAACCTGTGAAACAG
***SGPL1* EX6 for**	ATCCAGAGGAGTTTCTTCCT
***SGPL1* EX6 rev**	AAGGAGGTCATGTAAACTGG
***SGPL1* EX7 for**	ACTGTTGTTTAGTGCATGATTCT
***SGPL1* EX7 rev**	ACTGCAGTTAATTAGGATCTTTG
***SGPL1* EX8 for**	GAAATCGTGAGGATAGCTTG
***SGPL1* EX8 rev**	CACAATCTTCATCCCAAAG
***SGPL1* EX9 for**	GAACTTACTCCCGGTAATTTAGA
***SGPL1* EX9 rev**	GTCAGACCCATCTGACTGG
***SGPL1* EX10 for**	CTGGAACTCTAAGCTAGCAGC
***SGPL1* EX10 rev**	GAGCTACTTATCACTACTGTGGTCA
***SGPL1* EX11 for**	CATCTTTCCACCCATGTCT
***SGPL1* EX11 rev**	GTGACGGCAAAGAGAGAGT
***SGPL1* EX12 for**	TGCATGATGAGAGTTCTGG
***SGPL1* EX12 rev**	GAGACAACAGGTGGGCTA
***SGPL1* EX13 for**	GTGACCAGGGGATTGTATG

***SGPL1* EX13 rev**	TTGCTACTAACGTGCTAGCCT
***SGPL1* EX14 for**	CTTGTCAGAAATATTGTGAAAGG
***SGPL1* EX14 rev**	CAGACTCCGGGTCATATG
***SGPL1* EX15 for**	AGTGCACATGCGAAGCTA
***SGPL1* EX15 rev**	GAGGCTCAAGCTGTCTCAT
***SGPL1* cDNA for**	TGGAGATTTTGCATGGAG
***SGPL1* cDNA rev**	CACCTCCATCATCTTCGT
***SGPL1* cDNA for 2**	GATATCTTCCCAGGACTACG
***SGPL1* cDNA rev 2**	CATCATCTTCGTCAATGG
***GAPDH* cDNA for**	GAAGGTGAAGGTCGGAGTC
***GAPDH* cDNA rev**	GAAGATGGTGATGGGATTTC

Supplementary Table 6. Primer sequences for site directed mutagenesis

**Name**	**Sequence (5' to 3')**
***SGPL1* 'p.R222Q' for**	GCCTGCAAAGCATATCAGGATCTGGCCTTTGAG
***SGPL1* 'p.R222Q' rev**	CTCAAAGGCCAGATCCTGATATGCTTTGCAGGC
***SGPL1* 'p.F545del' for**	CAGAATTGTCCTCAGTCTTGGACAGCTTGTACAG
***SGPL1* 'p.F545del' rev**	CTGTACAAGCTGTCCAAGACTGAGGACAATTCTG

Supplemental Acknowledgements

GOSgene: We thank the members of GOSgene (Hywel Williams, Polona Le Quesne Stabej, Louise Ocaka, Chela James, Nital Jani, Chiara Bacchelli and Philip Beales. GOSgene is supported by the NIHR BRC at Great Ormond Street Hospital for Children NHS Foundation Trust and UCL Institute of Child Health.

Supplemental References

1. Baris S, Alroqi F, Kiykim A, Karakoc-Aydiner E, Ogulur I, Ozen A, Charbonnier LM, Bakir M, Boztug K, Chatila TA, et al. Severe Early-Onset Combined Immunodeficiency due to Heterozygous Gain-of-Function Mutations in STAT1. *Journal of clinical immunology.* 2016.
